# Real world study for the concordance between IBM Watson for Oncology and clinical practice in advanced non‐small cell lung cancer patients at a lung cancer center in China

**DOI:** 10.1111/1759-7714.13391

**Published:** 2020-03-19

**Authors:** Shuyang Yao, Ruotian Wang, Kun Qian, Yi Zhang

**Affiliations:** ^1^ Department of thoracic surgery Xuanwu Hospital, Capital Medical University Beijing China

**Keywords:** Advanced disease, artificial intelligence, concordance, non‐small cell lung cancer, Watson for Oncology

## Abstract

**Background:**

IBM Watson for Oncology (WFO) provides physicians with evidence‐based treatment options. This study was designed to explore the concordance of the suggested therapeutic regimen for advanced non‐small cell lung (NSCLC) cancer patients between the updated version of WFO and physicians in our department, in order to reflect the differences of cancer treatment between China and the United States.

**Methods:**

Retrospective data from 165 patients with advanced NSCLC from September 2014 to March 2018 were entered manually into WFO. WFO recommendations were provided in three categories: recommended, for consideration, and not recommended. Concordance was analyzed by comparing the treatment decisions proposed by WFO with the real treatment. Potential influenced factors were also analyzed.

**Results:**

Overall, the treatment recommendations were concordant in 73.3% (121/165) of cases. When two alternative drugs such as icotinib and nedaplatin were included as “for consideration,” the total consistency could be elevated from 73.3% to 90.3%(149/165). The logistic regression analysis showed that gender (*P* = 0.096), ECOG (*P* = 0.0.502), smoking (*P* = 0.455), and pathology (*P* = 0.633) had no effect on consistency, but stages (*P* = 0.019), including stage ≤III (77.8%, 21/27) and stage IV (93.5%, 129/138) had significant effects on consistency.

**Conclusions:**

In China, most of the treatment recommendations of WFO are consistent with the real world treatment. Factors such as patient preferences, prices, drug approval and medical insurance are also taken into consideration, and they ultimately affect the inconsistency. To be comprehensively and rapidly applied in China, localization needs to be accelerated by WFO.

## Introduction

Oncologists who treat lung cancer are challenged by a huge and rapidly expanding knowledge base. The growth of massive genetic and clinical databases will accelerate the speed of lung cancer treatment advances and shorten the cycle time for changes to lung cancer treatment guidelines. In 2019, up to September, NCCN non‐small cell lung cancer (NSCLC) guidelines had been updated five times.^1^ In addition, these information management challenges in cancer care are occurring in a practice environment where there is little time available for tracking and accessing relevant information at the point of care.

IBM Watson for Oncology (WFO) is one of the leading representatives in artificial intelligence (AI) or cognitive technologies. It is a unique system, with an ability to acquire much of its knowledge by “reading” the literature, protocols, and patient charts, and learning from test cases and experts from Memorial Sloan Kettering Cancer Center (MSKCC).^2^ It can identify connections among all of the data to answer a complex medical question in a very short amount of time, resulting in evidence‐based and personalized treatment options.^3^

Concordance studies have been performed in various countries. The results of a retrospective study focused on breast cancer in India indicated that 93% of WFO's recommendations for standard treatment or consideration were concordant with the recommendations of the tumor board.^4^ A study presented at the 2017 ASCO Annual Meeting of 525 patients from Korea showed a 73% concordance rate for colon cancer and a 49% concordance rate for gastric cancer.^5^ Although the studies are relatively limited, it has illustrated the differences in cancer therapy between the Eastern and Western world.

In fact, the use of WFO is becoming increasingly prevalent in China. WFO was introduced to China in March 2017 and has currently served more than 70 medical institutions nationwide above the city level and more than 10 000 patients.^6^ WFO was developed in the United States. Given the huge discrepancies in medical insurances and new drugs or therapies approved by individual food and drug agency (FDA) between two countries, many doctors and medical institutions in China have questioned whether WFO is suitable for Chinese cancer patients.

Our group aimed to explore the concordance of the suggested therapeutic regimen for advanced NSCLC patients between the updated version of WFO and physicians in our department, which could reflect the similarities and differences between the East and West in the treatment of cancer. Here, we report the results of this assessment and discuss the potential value of this technology as a learning system both for cases where concordance is found and where it is absent.

## Methods

### Study design

This study was approved by the ethics committee of Xuanwu Hospital, Capital Medical University.

### Watson for Oncology

WFO data are updated with the latest cutting‐edge information and verified by oncologists at MSKCC every 1–2 months. WFO version 19.2 was used in our study.

It is stated in the manual that WFO does not support certain cases. When we input a case not supported by WFO, the system will provide the reason as out of scope and no recommended treatment is returned. For supported cases, The treatment recommendations of WFO are categorized into three groups with a corresponding label: green represents “recommended treatments” with a strong base of evidence, amber represents treatments “for consideration” that oncologists may consider as suitable alternatives based on their clinical judgment, and red represents treatments that are “not recommended” due to specific contraindications or strong evidence against their use.^4^

WFO requires only the data of a case to be input, and within one minute, it generates patient‐specific treatment recommendation consistent with high quality evidence and MSKCC expertise.

### Patient selection

Data were collected on patients who received anticancer treatment in our center. The inclusion criteria for this study were as follows: (i) inpatients in our center; (ii) unresectable stage II, III or IV non‐small lung cancer patients who had not had radical resection; (iii) admission between September 2014 and March 2018, and (iv) received chemotherapies or targeted therapies. The exclusion criteria were as follows: (i) those who received only examinations and did not receive any antitumor treatment, and (ii) those who had just completed neoadjuvant therapy, then surgery, followed by adjuvant chemotherapy and no disease progression had been observed. A total of 165 cases were included in this study. The pathological types included squamous carcinoma, adenocarcinoma, and adenosquamous carcinoma.

### Operating procedure and evaluation of consistency

Patient information and actual treatments were collected from the electronic medical record system of our hospital: a senior physician who was blinded to the actual treatments manually input the patient information into WFO and recorded the decision by WFO for each patient, which was compared with the actual treatment by another doctor. If our actual therapeutic regimen was deemed recommended or for consideration by WFO, we defined the outcome as concordant, and if the actual treatment was deemed not recommended or not included in the recommendation by WFO, we defined the outcome as discordant. The team of specialists at our center reassessed the discordant cases and provided their reasons for choosing the actual regimens.

### Data analysis and statistics

We used Microsoft Excel and SPSS 22.0 to describe the data and to perform the statistical analysis. Descriptive statistics of the case characteristics were calculated using Microsoft Excel. Row percentages are presented where indicated. Concordance was expressed as percent agreement. Cancer characteristics included patient age, cancer stage, and *EGFR* gene mutation status. A logistic regression model was estimated with odds ratios and 95% confidence intervals (CIs).

## Results

### Characteristics of NSCLC cases

Demographic characteristics of the enrolled NSCLC patients are shown in Table 1. Patients had a median age of 61 years, of whom 66.1% (109/165) were males and 33.9% (56/165) were females. Adenocarcinoma with epidermal growth factor receptor (*EGFR*) gene mutation accounted for 38.0% (46/121), and phase IV disease accounted for 83.6% (138/165) of all patients. Most of the patients had an ECOG score of 1 (115/165).

**Table 1:** Characteristics of the NSCLC cases (*n* = 165)

Characteristics	Results
Gender, *n* (%)	
Male	109 (66.1)
Female	56 (33.9)
Pathology, *n* (%)	
Squamous carcinoma	43 (26.0)
Adenocarcinoma	121 (73.3)
Adenosquamous carcinoma	1 (0.7)
Smoking, *n* (%)	
Yes	70 (42.4)
No	95 (57.6)
Stage, *n* (%)	
≤III	27 (16.4)
IV	138 (83.6)
ECOG status	
0	50 (30.3)
1	115 (69.7)
Epidermal growth factor receptor (*EGFR*) gene mutation status, *n* (%)	
*EGFR* mutation	43 (26.1)
Wild‐type *EGFR*	122 (73.9)

### Concordance of supported cases and the influencing factors

**Figure 1:**
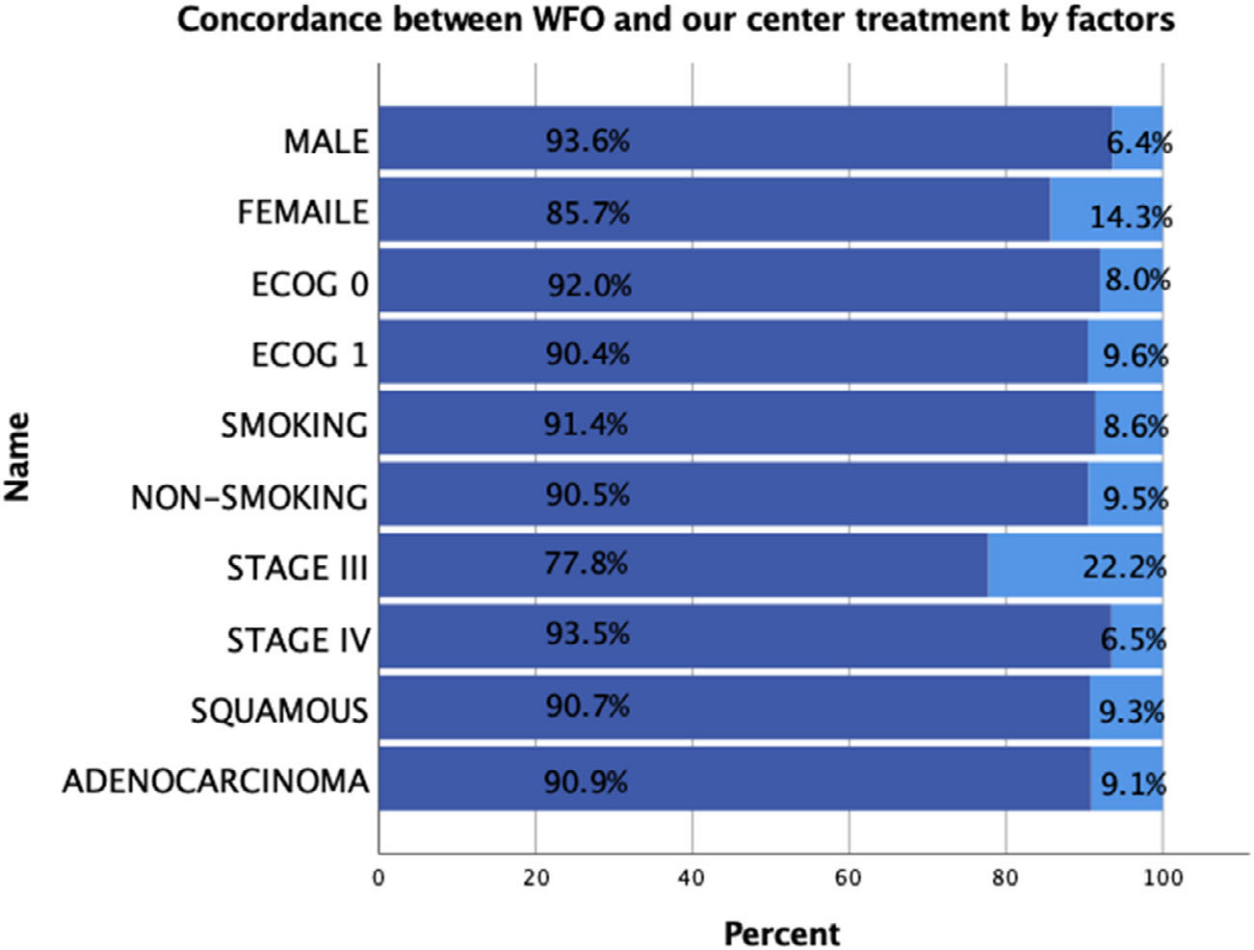
Concordance between WFO and our center treatment by gender, ECOG, smoking, stage and pathology. Concordance (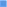) inconsistency and (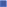) consistency.

Overall, the treatment recommendations were concordant in 73.3% (121/165, shown in Table 2) of cases. The concordant rate in males was 93.6%, and in females was 85.7%; ECOG = 0 was 92.0%, and ECOG = 1 was 90.4%; smoking was 91.4%, and non‐smoking was 90.5%; Stage ≤III was 77.8%, and stage IV was 93.5%; squamous was 90.7%, adenocarcinoma was 90.9% (shown in Fig 1).

**Table 2:** Logistic regression model of concordance between Watson for Oncology and treatment data

Characteristics	Odds ratio (95% CI)	*P*‐value
Gender	2.429 (0.832–7.086)	0.096
ECOG	0.822 (0.249–2.718)	0.502
Smoking	1.529 (0.498–4.692)	0.455
Pathology	1.000 (0.301–3.321)	0.633
Stage	4.095 (1.321–12.694)	0.019

To explore the reason for discordance, we found that there were 18 patients treated with icotinib and 10 patients with nedaplatin considered as discordant cases. These drugs are approved by China's FDA and supported by Chinese guidelines for its use in advanced NSCLC. However, they have not yet been approved by the FDA in the United States and thus were not included in the WFO. If we replaced them with two alternative treatments with similar effectiveness available in WFO (icotinib to gefitinib, nedaplatin to cisplatin), the adjusted concordance could elevate from 73.3% to 90.3% (149/165).

There were another 15 patients whose treatments were assessed as “not recommended” or “not available” by WFO. Among these, five patients had not received WFO recommended chemoradiotherapy and nine patients had received monodrug chemotherapy, instead of the WFO recommended double agent regimen as the first‐line treatment, and there was one patient with stage IIb whose tumor had not been surgically resected.

Logistic regression analysis showed that gender (*P* = 0.096), ECOG (*P* = 0.0.502), smoking (*P* = 0.455), and pathology (*P* = 0.633) had no significant effect on concordance stages (*P* = 0.019), had significant effects on consistency (shown in Table 2), and the concordance in stage IV (93.5%) was substantially higher than in stage ≤III (77.9%, Fig 1).

## Discussion

This study is the first retrospective study to explore the concordance between an AI‐based clinical decision support system and the real world decision in Chinese advanced NSCLC patients. With regard to WFO and native guidelines, the overall concordance is high with the leading lung cancer center in China, suggesting the potential impact of WFO in minimizing the medical disparities across different regions of China.

In this retrospective study, the number of cases where our actual treatments were in the “recommended treatments” category of WFO was only five. This is mainly because the patients were enrolled from 2014 to 2018, but the WFO version used in this study was 19.2, which was updated in May 2019. The differences between recruitment of patients and input time of WFO are closely related to the great advances which have been made in lung cancer treatment. Immunotherapy with PD‐1/PD‐L1 antibody drugs have been approved in the U.S. since 2014, and WFO recommend treatment with immunotherapy drugs such as pembrolizumab, or nivolumab and either one alone or combined with chemotherapy for metastatic NSCLC as the first‐line treatment, but chemotherapy was the only regimen available for consideration. Since the FDA in China had not approved these immunotherapy drugs until last August, at that time we had to select chemotherapy. Similarly, osimertinib is the only recommended first‐line treatment in WFO for *EGFR* sensitive mutant patients, but even now in China osimertinib is approved and covered by medical insurance in some limited provinces only for T790M (+) EGFR patients. Therefore, we could only use other EGFR TKIs such as gefitinib or icotinib which were ranked as for consideration options in WFO. In this study, WFO did not support 9.1% (15/165) of the cases. There is a great difference in the *EGFR* gene mutation phenotype of lung cancer in China compared with that in Western countries. The *EGFR* mutation rate of lung cancer in European and American countries is approximately 15%. In this study, the mutation rate was 35.5% in adenocarcinoma and 26.1% in the whole group. The treatment consistency was 73.3% (121/165), which was much lower than 96.4% reported in an abstract at the 2017 American Society of Clinical Oncology Annual Meeting.^7^

Nonconcordance was due to the availability of therapies in China that were not included in the US‐trained oncology advisor. First, WFO recommends concurrent chemoradiation for stage III, but meanwhile in China clinicians mostly perform sequential chemoradiation (chemotherapy followed by radiotherapy) due to the lower tolerability of Chinese patients. Second, China uses the drugs icotinib and nedaplatin instead of the other first‐generation drugs EGFR‐TKI and cisplatin or carboplatin (16/165, 10/165, respectively). Icotinib is a primary research drug in China, and studies have shown that it is as effective as the other EGFR‐TKI first‐generation drugs.^6^, ^7^, ^8^ Compared with cisplatin, nedaplatin, a cisplatin derivative, has fewer toxic effects, higher response rate and longer overall survival, especially in patients with squamous carcinoma.^9^, ^10^ If WFO was able to output these two alternative treatments as “recommended” or “for consideration,” the overall consistency could be elevated from 73.3% to 90.3%. Third, some patients' personal status were 1 but they were around 80‐years‐old. Their organ functions are definitely worse than younger patients, and it was considered that the potential for them to suffer from treatment with platinum‐based chemotherapy was high. In clinical work, they had received monodrug chemotherapy such as gemcitabine for lung squamous carcinoma and pemetrexed for adenocarcinoma.

Moreover, as mentioned in the manual, WFO does not support certain cases. In this study, WFO did not support 9.1% (15/165) of the cases. In our opinion, the possible reasons are that these cases are relatively personalized and complicated, lacking in substantial evidence and training cases. For example, in our study, a 54‐year‐old patient, diagnosed with stage IIb lung adenocarcinoma, had just recovered from a severe pneumonia, making her respiratory function too poor to survive radical surgery. Therefore, our treatment strategy was to prescribe pemetrexed combined with carboplatin.

Until now, the published literature on WFO in advanced non‐small cell lung cancer has been very limited. The study by Liu *et al*. on WFO in lung cancer patients showed the general consistency was 65.8% and after localization, the overall consistency was elevated to 93.2%.^6^ The results are very similar to those in our study. It should be highlighted that in this study, the rate of EGFR status unknown patients was 65.1% and *EGFR* mutation was only 5.4%, which was much lower than our study, even lower than the EGFR positive rate in the overall NSCLC group. Small cell lung cancer was also included in their study. It is well known that the treatment gap between western and eastern countries is narrow, and the consistency in this group was much higher than NSCLC. In another study, 113 lung cancer patients were included and 78% were NSCLC, the concordance of NSCLC was 79.99%, but there was not much detailed analysis in this study.

As AI development has accelerated since 2015, it has been introduced to empower clinical decision support systems (CDSS) for different applications in the medical domain, including diagnostics, population health management, therapeutics and administration.^8^ Furthermore, CDSS, integrated with electronic medical records is one of the major branches of application by using natural language processing (NLP) and machine learning to generate instruction on standardized practice and rational drug use, which helps decrease the inappropriate medication rate by approximately 15%–30%.^9^ Being the representative of application‐level NLP‐based CDSS, WFO has several unique advantages. First, WFO is verified and endorsed by MSKCC, guaranteeing its safety and reliability proved by the comparison with top tier hospitals in different regions.^3^, ^4^, ^6^ Moreover, WFO provides evidence extracted from a great number of relevant literature such as clinical trials and related studies to make its recommendations interpretable and acceptable to clinicians. Therefore, clinicians are more inclined to learn from and refer to WFO to improve the quality of their decisions.^10^ By applying WFO in a clinical scenario, the healthcare providers in less developed regions can possibly deliver cancer care of standardized and neutral quality, eliminate bias of individuals, and save patients valuable time in having to visit multiple top hospitals just for a second opinion.

However, AI enabled CDSS still faces obstacles before it can reach widespread use in clinical medicine. With clinical decision‐making, there is often no gold standard or single “right answer,” and decisions usually involve social and psychological factors beyond linkages between clinical data and medical knowledge reflected by a CDSS.^5^ On the other hand, it is noteworthy that WFO only stores established knowledge which is supported by evidence and verified by MSKCC. Nonetheless, if scientists try to use AI to obtain new insights from data other than established knowledge, they will probably find it extremely difficult to interpret and evaluate the output, namely the “black box” problem, which limits the quick deployment of AI‐CDSS in clinical medicine.^11^ Other challenges involve concerns about patient privacy, security^12^ and extra payment for WFO.

Our study has several important strengths. First, it includes a large number of advanced NSCLC patients and compares the recommendation of WFO to that of a lung cancer center, which belongs to a large comprehensive hospital. Second, the physicians and WFO were blinded to each other's treatment recommendations, eliminating potential influence by the other's previous assessment. Third, we report the consistency of the recommendations from WFO with those from our cases, and we have analyzed the potential factors and provided some suggestions for the improvement of WFO which would be better suited to Chinese patients.

There are several notable limitations in our study. First, this study is a retrospective study without a control group, making the results potentially vulnerable to the influence of unmeasured factors. Second, the time discrepancy difference between the original tumor assessments and the later WFO review was addressed. Many new drugs have been approved by the Food Drug Administration (FDA) in the last few years and treatment strategies have subsequently been updated. The discrepancies between both nations' FDA has also affected the recommendations of WFO. Third, the bias of input data from experts would be expected to cause lower quality recommendations by WFO and to reduce concordance.

In conclusion, WFO is a good tool to assist Chinese oncologists and a good tutorial for young physicians; it is also helpful in its standardization of the treatment of lung cancer in China. However, it remains essential to build our national medical data library to improve WFO so that it is well localized which will enable us to greatly improve the treatment of Chinese lung cancer patients.

## Disclosures

The authors declare that there are no conflicts of interest.
